# Three-dimensional skeleton networks of graphene wrapped polyaniline nanofibers: an excellent structure for high-performance flexible solid-state supercapacitors

**DOI:** 10.1038/srep19777

**Published:** 2016-01-22

**Authors:** Nantao Hu, Liling Zhang, Chao Yang, Jian Zhao, Zhi Yang, Hao Wei, Hanbin Liao, Zhenxing Feng, Adrian Fisher, Yafei Zhang, Zhichuan J. Xu

**Affiliations:** 1 Key Laboratory for Thin Film and Microfabrication Technology of the Ministry of Education, School of Electronics, Information and Electrical Engineering, Shanghai Jiao Tong University, Dong Chuan Road No. 800, Shanghai, 200240, P. R. China; 2 School of Materials Science & Engineering, Nanyang Technological University, 50 Nanyang Avenue Block, 639798, Singapore; 3 Solar Fuels Lab, Nanyang Technological University, 50 Nanyang Avenue, Singapore 639798, Singapore; 4 Chemical Science and Engineering Division, Argonne National Laboratory, Argonne, IL, 60439, United States; 5 Department of Chemical Engineering, Cambridge University, Cambridge CB2 3RA, United Kingdom

## Abstract

Thin, robust, lightweight, and flexible supercapacitors (SCs) have aroused growing attentions nowadays due to the rapid development of flexible electronics. Graphene-polyaniline (PANI) hybrids are attractive candidates for high performance SCs. In order to utilize them in real devices, it is necessary to improve the capacitance and the structure stability of PANI. Here we report a hierarchical three-dimensional structure, in which all of PANI nanofibers (NFs) are tightly wrapped inside reduced graphene oxide (rGO) nanosheet skeletons, for high-performance flexible SCs. The as-fabricated film electrodes with this unique structure showed a highest gravimetric specific capacitance of 921 F/g and volumetric capacitance of 391 F/cm^3^. The assembled solid-state SCs gave a high specific capacitance of 211 F/g (1 A/g), a high area capacitance of 0.9 F/cm^2^, and a competitive volumetric capacitance of 25.6 F/cm^3^. The SCs also exhibited outstanding rate capability (~75% retention at 20 A/g) as well as excellent cycling stability (100% retention at 10 A/g for 2000 cycles). Additionally, no structural failure and loss of performance were observed under the bending state. This structure design paves a new avenue for engineering rGO/PANI or other similar hybrids for high performance flexible energy storage devices.

The rapid development of next-generation flexible electronics stimulates the urgent demand for flexible power sources^1^,^2^. Supercapacitors (SCs), as one of remarkable energy storage devices, have recently received great attention for powering electronics because of their high power capability, ultra-long cycling life, and low maintenance cost^3^. Various electrode materials, such as carbon nanomaterials^4^, metal oxides^5^, and conducting polymers^6^, etc., have been developed for SCs. Graphene, an intriguing two-dimensional honeycomb carbon sheet, has been recognized as an ideal electrode material^7^ due to its unique properties, including high Young’s modulus, large specific surface area, and excellent conductivity, etc.^8^ Graphene sheets could be prepared in a large quantity through the reduction of graphene oxide (GO)^9^. They have been extensively investigated as electrode materials and exhibited extraordinary electrochemical performance outperforming many other carbon materials^10^,^11^. More excitingly, flexible rGO films with compact or three-dimensional (3D) structures can be formed in a large scale through assembly of GO nanosheets, which enables rGO films as one of ideal candidates for flexible SCs^12^,^13^. However, the pure rGO-based electrodes usually give relatively low specific capacitance due to the limited capacitance contribution from the double layer storage mechanism^10^. Thus, developing composite electrodes through hybridization of rGO with pseudocapacitive materials, such as metal oxides and conducting polymers, has become popular for high-performance flexible SCs^13^. It greatly enhances the energy density and also alleviates the poor stability and slow response of pseudocapacitive materials during the charge-discharge cycles^13^.

Polyaniline (PANI), a conjugated conducting polymer, has been considered as one of the most promising pseudocapacitive electrode materials due to its facile synthesis, high reliability, high multiple redox-states-derived specific pseudocapacitance, and low cost^14^. However, PANI usually suffers from the volume change and structure destruction during the insertion/deinsertion process of the counter ions, leading to a poor cycle life^15^. Hybridization of PANI with rGO has been widely investigated and it has been found that the enhancement on the electrochemical performance depends greatly on the synthesis method^16^,^17^,^18^,^19^,^20^,^21^,^22^,^23^. This is because the enhancement relies on the combination of electric double layer capacitive rGO and pseudocapacitive PANI as well as the hybridization-derived synergistic effects^15^.

In general, the microstructure of electrode materials plays critical roles in the performance of SCs^10^. Various methods, such as *in situ* polymerization^16^, tailoring nanostructured PANI on rGO sheets^17^,^18^,^19^, and grafting PANI with graphene^20^, etc., have been reported to fabricate powdery rGO/PANI electrode materials. However, the powder-based electrode materials have to mix with insulating binders, which inevitably affect the electrical conductivity and thus the overall capacitance is compromised^21^. In addition, it is quite challenging to immobilize powdery materials on electrode substrates especially under bending state. Hence, it’s highly desired to develop flexible high performance graphene-PANI film-type electrodes. Several reports have revealed that the ordered structure of PANI and the intimate contact between PANI and rGO can improve the performance of the hybrid electrodes^15^,^22^,^24^. The electrochemical deposition technique has been found effective for making the ordered structures and so-fabricated hybrid film electrodes have shown excellent performances^14^,^15^,^23^. However, few of these methods work well for solid-state flexible SCs^25^,^26^,^27^ in terms of both areal and volumetric energy densities. The great challenges remain in fabricating high-performance flexible solid-state SCs, with high areal and volumetric energy storage capability, superior electron and ion conductivity, robust mechanical flexibility, as well as long term stability.

Herein, we report for the first time a facile method to fabricate a unique 3D structure, constituting from porous hydrogel films of continuous rGO nanosheet network skeletons embedded with PANI nanofibers (NFs) for high-performance solid-state SCs. PANI NFs can be tightly wrapped by rGO nanosheets and subsequently embedded inside rGO nanosheet network skeletons. This design not only avoids the stacking of rGO nanosheets, but also ensures all of rGO nanosheets directly interconnected with each other to form ideal conductive networks. Our designed rGO-PANI NFs 3D structure exhibits highest gravimetric specific capacitance of 921 F g^−1^ and volumetric capacitance of 391 F cm^−3^ at a discharge current density of 1 A g^−1^ as compared to all graphene-PANI hybrid film-based electrodes reported to date. These film electrodes are further assembled to build the flexible solid-state SC, which shows a high areal capacitance of 0.9 F cm^−2^ and a competitive volumetric capacitance of 25.6 F cm^−3^. The SC delivers a high areal energy density of 0.13 mWh cm^−2^ (2.1 mW cm^−2^ at 4.2 mA cm^−2^) and a high volumetric energy density of 3.6 mWh cm^−3^ (0.06 W cm^−3^ at 0.12 A cm^−3^), showing outstanding rate capability (~75% retention at 20 A g^−1^) and excellent cycling stability (>100% retention at 10 A g^−1^ for 2000 cycles) under bending state without structural failure and performance loss.

## Results

**Figure 1:**
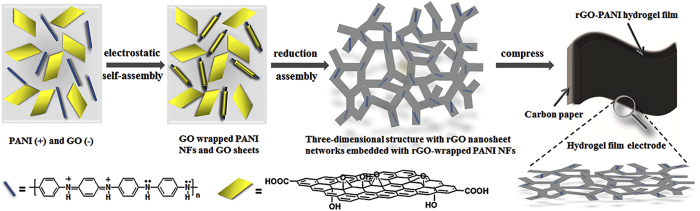
Schematic illustration of the preparation of the flexible hybrid hydrogel film electrodes with continuous three-dimensional rGO nanosheet network skeletons embedded with rGO-wrapped PANI NFs.

**Figure 2:**
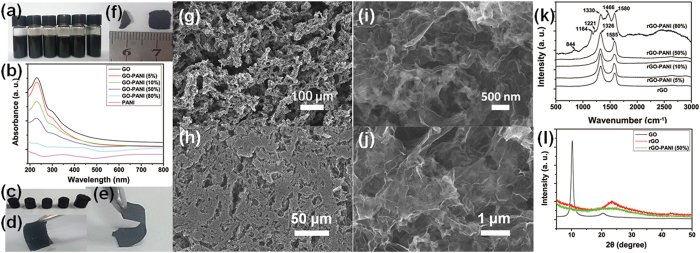
Characterization of as-synthesized materials. (**a**) Digital photographs of aqueous dispersions of PANI NF, GO, GO-PANI NF hybrids with different contents of PANI (5 wt%, 10 wt%, 50 wt%, and 80 wt%) from left to right. (**b**) UV-Vis spectra of aqueous solutions of GO, PANI, and GO-PANI hybrids. Photographs of (**c**) the corresponding rGO and rGO-PANI hybrid hydrogels, (**d**) rGO-PANI (50%) hydrogel films on carbon papers, (**e**) Free-standing freeze-dried rGO-PANI (50%) hydrogel films, and (**f**) free-standing air-dried rGO and rGO-PANI (50%) hydrogel films. Low- and high-magnification SEM images of interior microstructures of (**g,i**) freeze-dried rGO-PANI (50%) hybrid hydrogels and **(h,j**) the corresponding freeze-dried hydrogel films. (**k**) Raman spectra of the as-prepared freeze-dried rGO and rGO-PANI NF hybrid hydrogel films. (**l**) XRD patterns of GO, the freeze-dried rGO and rGO-PANI (50%) hybrid hydrogel films.

The schematic illustration in Fig. 1 shows the preparation of rGO-PANI NF hybrid hydrogel film electrodes. The hybrid hydrogels, with different weight ratios of PANI NFs to GO, were prepared through hydrothermal reduction of GO by L-cysteine. To design the rGO nanosheets-wrapped PANI NF structures, the electrostatic self-assembly was allowed to take place in aqueous solution before hydrothermal treatments. It is known that single GO nanosheets possess myriads of negative –COOH groups on the surface^28^, which enables GO to be dispersed in water efficiently and facilitates self-assembly of GO onto the surface of PANI NFs. In order to ensure the efficient dispersion of PANI NFs, the pH of the solution was adjusted to ~4. The resulting PANI NFs had a net positive charge due to the inherent structure^29^. Hence the electrostatic stabilization was realized^30^. The obtained PANI NF aqueous colloid can be stable for more than one week (Fig. 2a). During electrostatic assembly process, the weak acid solution enabled the dispersion of positively charged PANI NFs and prevented the agglomeration of negatively charged GO nanosheets. The resultant GO-PANI NF hybrid suspensions with different PANI NF contents were stable for more than one week (Fig. 2a). The TEM images suggested that almost all of PANI NFs have been wrapped by GO nanosheets for GO-PANI hybrids with the content of PANI NFs lower than 50% (Supplementary Fig. S1a–c). In contrary, GO-PANI hybrids with 80% PANI NFs showed that lots of PANI NFs were distributed between stacked GO nanosheets (Supplementary Fig. S1d). From Fig. S1, lots of free GO nanosheets can be seen in all GO-PANI hybrid suspensions, which benefits the hydrogel formation through the subsequent hydrothermal assembly process. The wrapping was also confirmed by UV-Vis spectra as shown in Fig. 2b. The GO-PANI hybrids with the contents of PANI NFs lower than 50% exhibited similar absorption peaks, at 229 and 298 nm, with characteristic of pure GO colloids. Meanwhile, GO-PANI (80%) dispersions showed a similar absorption behavior with pure PANI NFs. It suggests that wrapping of PANI NFs with GO nanosheets had taken place for GO-PANI hybrids with the content of PANI NFs lower than 50%, which shielded the characteristic peaks of PANI NFs. It indicates the formation of the final rGO nanosheets skeletons intimately embedded with rGO-wrapped PANI NFs. Using GO-PANI hybrids as precursors, hydrothermal reduction reactions were carried out and the hydrogels were formed for all rGO-PANI hybrids, as shown in photographs of Fig. 2c. Compared with pure rGO hydrogels, the mass loadings of PANI NFs in rGO-PANI hybrid hydrogels were 11%, 20%, 69%, and 90%, respectively, for rGO-PANI (5%), rGO-PANI (10%), rGO-PANI (50%), and rGO-PANI (80%) hydrogels (Supplementary S1.3). Brunauer-Emmett-Teller (BET) and Barrett-Joyner-Halenda (BJH) analysis as well as the methylene blue dye adsorption method^31^ revealed that the freeze-dried rGO and hybrid hydrogels had fairly high specific surface areas from 375 to 585 m^2^ g^−1^ and the pore sizes in the range of 2–50 nm (Supplementary S2.1 and Fig. S2).

The hydrogel films were then fabricated by pressing thin hydrogel slices on carbon papers. The wet hydrogel films were able to attach on carbon papers (as current collector) firmly and directly used as electrodes for further testing and device assembling (Fig. 2d). The freeze-dried hydrogel films were spontaneously peeled off from carbon paper. The hydrogel films with the contents of PANI NFs lower than 50% were robust for flexible SCs (Fig. 2e), while rGO-PANI (80%) hydrogel film was very brittle. The rGO hydrogel film and rGO-PANI (50%) hydrogel film were also dried at room temperature. It’s interesting to see that the size of rGO-PANI (50%) hydrogel film barely changed, whereas the rGO hydrogel film shrunk significantly (Fig. 2f), indicating synergistic interactions occurred between rGO nanosheets and PANI NFs. The microstructures of rGO-PANI (50%) hydrogel before and after pressing were observed by SEM (Fig. 2g–j). Figure 2g,i revealed that the rGO-PANI (50%) hydrogel had interconnected 3D macro-porous networks with pore sizes ranging from sub-micrometers to several micrometers and pore walls of worm-like rGO-PANI NF hybrids. After pressing, the resultant hydrogel film showed a more compact structure with majority of macro-pores fused. Interestingly, the microstructures of the films didn’t change (Fig. 2h,j). Figures S3 and S4 also confirmed that all of PANI NFs were embedded in rGO networks when the contents of PANI NFs were lower than 50%. It can be seen that a large quantity of PANI NFs on the surfaces of rGO networks for rGO-PANI (80%), suggesting that PANI NF content was the key for the wrapping process.

Raman spectra of rGO-PANI hybrid hydrogel films with the contents of PANI NFs lower than 50% in Fig. 2k exhibited a D band and a G band peaks similar with characteristic of rGO^28^. The rGO-PANI (80%) hydrogel film showed a series of peaks similar with characteristic of PANI^15^, such as C-H bending of quinonoid and benzene rings at 844 and 1164 cm^−1^, the semi-quinone radical vibration at 1330, 1466, and 1580 cm^−1^, and the C-N stretching mode of the polaronic units at 1221 cm^−1^. It suggests ideal wrapping of PANI NFs by rGO nanosheets occurred when the contents of PANI NFs were lower than 50%. This result is consistent with TEM, UV-Vis absorption, and SEM studies, indicating the success of the construction of 3D structures with rGO sheet network skeletons embedded with rGO-wrapped PANI NFs.

In addition, XRD patterns (Fig. 2l) confirmed the efficient de-oxygenation of GO to form rGO upon L-cysteine reduction. A broader XRD peak of rGO-PANI (50%) hydrogel film is a sign of the poorer ordering for rGO sheets along their stacking direction due to the existence of PANI NFs^31^. Moreover, Fig. S5 revealed that the characteristic peak of hybrid hydrogel films became broader as the increase of contents of PANI NFs. The broad peaks of all hybrid hydrogels further reflected that the skeletons of hydrogels were composed of few-layer stacked rGO nanosheets embedded with PANI NFs. The reduction of GO and existence of PANI in the hybrid hydrogels were also confirmed by XPS and FTIR (Supplementary S2.2 and Figs S6 and S7).

## Discussion

**Figure 3:**
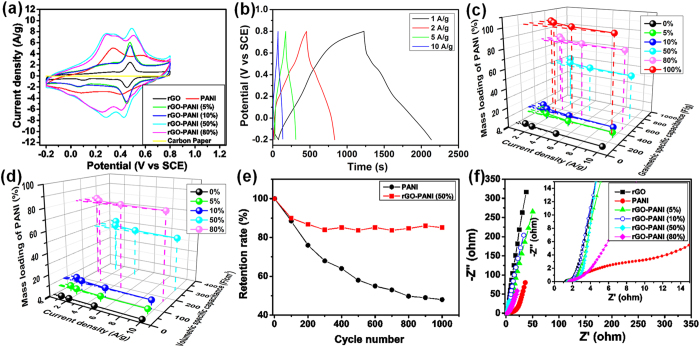
Electrochemical performance of as-fabricated single electrodes. (**a**) CV curves of Carbon paper and PANI NF, rGO-, and rGO-PANI NF hybrids-based hydrogel film electrodes at a scan rate of 5 mV/s. (**b**) Galvanostatic charge/discharge curves of rGO-PANI (50%) hybrid hydrogel film electrodes at different current densities. (**c**) Comparison of gravimetric specific capacitances of the as-prepared electrodes with different mass loadings of PANI at different current densities. (**d**) Comparison of the volumetric specific capacitance of the as-prepared hydrogel film electrodes with different mass loadings of PANI at different current densities. (**e**) Cycling stability of the PANI NF and rGO-PANI (50%) hybrid hydrogels film-based electrodes at a current density of 10 A/g. (**f**) Nyquist plots of PANI NF, and rGO and rGO-PANI NF hybrids hydrogel film electrodes. The inset shows the corresponding magnified high-frequency regions.

As describe above, it will be interesting to study the electrochemical performances of as-fabricated film electrodes with this unique structure. Figure 3a shows the CV curves of PANI, rGO and rGO-PANI hybrids hydrogel film electrodes at a scan rate of 5 mV s^−1^. The CV curves of rGO hydrogel films exhibit a pair of redox peaks. This is probably due to the existence of L-cystine residues formed from L-cysteine during reduction process of GO^32^, which was also confirmed by existence of S species in XPS survey spectra as shown in Fig. S6c. As for PANI and all rGO-PANI hybrid films, two couples of redox peaks appear, which can be ascribed to the leucoemeraldine/emeraldine and emeraldine/pernigraniline transition of PANI^15^. The hybrid hydrogel films show larger integrated areas than that of the rGO hydrogel film, revealing the large pseudocapacitance behavior of PANI^33^. CVs of all electrodes at different scan rates from 5 to 100 mV s^−1^ were shown in Fig. S8. It’s noted that the cathodic peaks of all electrodes shift positively and the anodic peaks shift negatively at higher scan rates. It should be caused by the internal resistance of electrodes^33^. The hybrid hydrogel films show similar shape variations with rGO hydrogel film except for rGO-PANI (80%) hydrogel film, suggesting that wrapping of PANI NFs by rGO nanosheets can ensure the formation of excellent conducting networks of interconnected rGO nanosheets and the achievement of good rate capability of the final electrodes. Moreover, rGO-PANI (50%) hybrid hydrogel film has the largest integrated area in CV curves at the same scan rate (Fig. 3a and Supplementary Fig. S8). It suggests that the synergistic interaction between PANI NFs and rGO nanosheets has occurred in rGO-PANI (50%) hydrogel film, leading to much higher specific capacitance than that of rGO hydrogel film, PANI electrode materials, and other hybrid hydrogel films.

The GCD curves of all electrodes show a deviation from the ideal triangular shape (Fig. 3b and Fig. S9), indicating the significant contribution of pseudocapacitance. The largest specific capacitance at 1 A g^−1^ of the rGO-PANI (50%) hydrogel film can be observed in GCD, which is consistent with the CV result (Fig. 3a). In order to better compare the capacitance of these electrode materials with different PANI mass loadings, the specific capacitance values were derived from GCD curves of all electrodes (Fig. 3b and Supplementary S1.4.1 and Fig. S9) and shown in Fig. 3c. With the increase of PANI contents from 0% to 100% (based on the mass of GO and PANI), the gravimetric specific capacitance of the resultant electrodes firstly increased and then decreased. A high specific capacitance value of 921 F g^−1^ at 1 A g^−1^ can be obtained by rGO-PANI (50%) hydrogel film, much higher than that of rGO hydrogel film (128 F g^−1^) and PANI NFs (575 F g^−1^). Note that this value is the highest among all rGO-PANI hybrid film electrodes reported to the best of our knowledge (Supplementary Table S1). It suggests that the unique 3D structures leads to a synergistic effect between rGO sheets and PANI NFs. Based on the specific capacitance of rGO hydrogel film and PANI NFs, the enhanced capacitance of rGO-PANI (50%) hydrogel film contributed by synergistic effect is about 485 F g^−1^ (Supplementary S1.4.2), reaching 52.7% of that of the hybrid hydrogel film, revealing the key role of synergistic effect in the novel 3D structure on improving the final capacitive behavior^15^. In Fig. 3c, the increase of current density results in the decrease of specific capacitance for all electrodes and the rGO-PANI (50%) hydrogel film showed the best performance. Upon increasing the current density up to 2 and 5 A g^−1^, the specific capacitance of rGO-PANI (50%) hydrogel film remained at 771 and 701 F g^−1^, with the capacity retention of 84% and 76%, respectively. Even at a high current density of 10 A g^−1^, the specific capacitance of the electrode still remained at 655 F g^−1^, 71% of that at 1A g^−1^, about 5.8 and 1.4 times higher than that of rGO hydrogel film (95 F g^−1^) and PANI NFs (266 F g^−1^), respectively, indicating the excellent rate capability of rGO-PANI (50%) hydrogel film for high-performance SCs.

Volumetric specific capacitance is one of the most important performance metrics for electrodes^34^,^35^,^36^. The corresponding results are calculated and shown in Fig. 3d. A volumetric capacitance of 391 F cm^−3^ at 1 A g^−1^ (i.e., 0.42 A cm^−3^) is obtained by rGO-PANI (50%) hydrogel film, which is much higher than that of rGO hydrogel film (19.4 F cm^−3^) and other hybrid hydrogel film electrodes (Fig. 3d).

The long-term cycling stability of rGO-PANI (50%) hydrogel film was examined and compared with that of PANI electrode using consecutive GCD cycles at a current density of 10 A g^−1^ (Fig. 3e). The rGO-PANI (50%) hydrogel film showed excellent stability with 85.2% of its initial capacitance retained after 1000 cycles, while PANI NFs kept only 48% of its capacitance. It’s believed that the improved stability of rGO-PANI (50%) hydrogel film came from the synergistic effect of rGO sheets and PANI NFs. The continuous and flexible rGO nanosheet network skeleton not only offered a better conductivity, but also helped maintain the structures of PANI during insertion/de-insertion process, which is critical for achieving a better stability of the hybrid hydrogel film electrode. This is further supported by SEM analysis on rGO-PANI (50%) hydrogel film and PANI NF electrode after cycling (Fig. S10). PANI NFs of the hybrid hydrogel film electrode were well incorporated in rGO sheet networks, suggesting PANI NFs were protected well by rGO nanosheets, while the compact structure of the PANI NF electrode (Supplementary Fig. S10c and d) were destroyed after cycling (Supplementary Fig. S10e and f). This SEM comparison highlights the key role of rGO nanosheets on protection of PANI. The Nyquist plot shown in Fig. 3f further revealed the excellent electrical conductivity of the hybrid film electrodes over the pure PANI NF electrode. Different from rGO-PANI (80%) hydrogel film electrode, rGO-PANI (50%) hydrogel film showed almost a straight line in the low-frequency region similar with rGO hydrogel film. In the high-frequency region, rGO-PANI (50%) hydrogel film showed an inconspicuous semicircle, which is related to the reduced electrical charge transfer resistance, suggesting a very low charge transfer resistance^37^.

It’s worth noting that the gravimetric and volumetric specific capacitances of rGO-PANI (50%) hydrogel film electrode are significantly higher than those of previously reported graphene-PANI hybrid film electrodes, SCs, and many other graphene-based film electrodes, as listed in Table S1 and S2. A fairly high cycling stability has also been obtained. Considering the high current density of 10 A g^−1^, much higher than those of other rGO-PANI electrodes, is applied for cycling in this work, the rGO-PANI (50%) hydrogel film electrode is more attractive for high-performance SCs. It’s proposed that the outstanding performance of the hybrid hydrogel film electrodes is due to the unique 3D structure of the hybrids. First, the efficient dispersion of PANI NFs and self-assembled GO-PANI hybrids ensured the formation of porous rGO-PANI hybrid hydrogels, in which the 3D rGO nanosheet network skeleton provides a large surface area for accommodating PANI NFs. This greatly enhanced pseudocapacitance. Second, PANI NFs were wrapped by rGO nanosheets, resulting in an intimate contact between rGO nanosheets and PANI NFs. This wrapping not only protected PANI during cycling but also offered rapid electron transfer between PANI NFs and rGO nanosheet networks. Third, the embedment of rGO-wrapped PANI NFs inside rGO nanosheet network skeletons avoided the stacking of rGO nanosheets and ensured all of rGO nanosheets to be interconnected with each other to form ideal conductive networks. Finally, the interconnected rGO nanosheets-wrapped PANI NFs skeletons have a large group of micro- and macro-porous structures, which can facilitate ions diffusion into the interior pores and efficient electron transport throughout the entire 3D framework.

**Figure 4:**
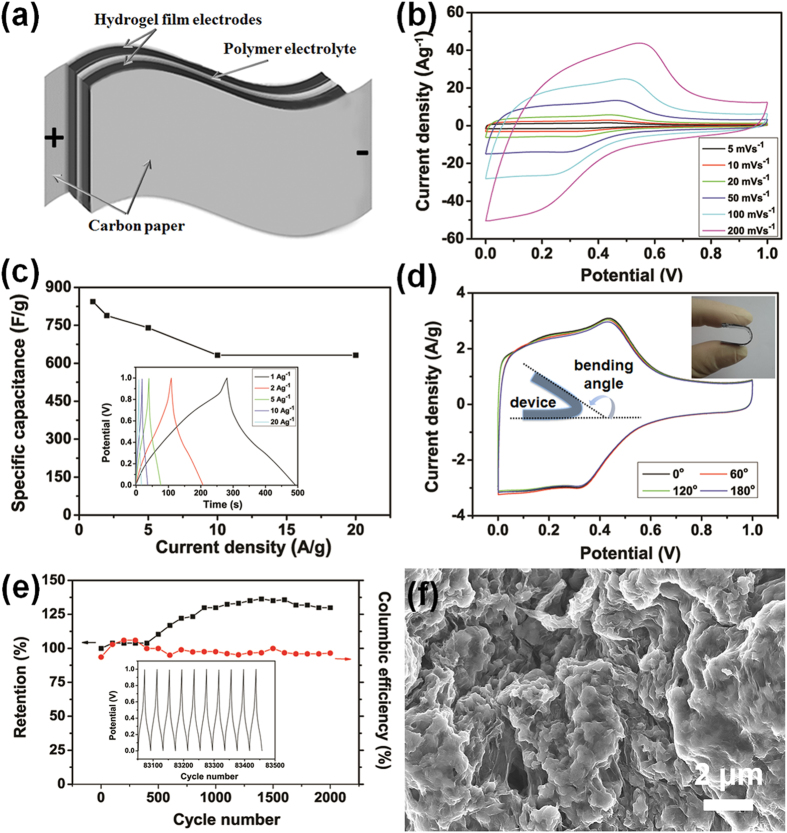
Electrochemical performance of as-fabricated flexible devices. (**a**) A schematic diagram of the solid-state device with H_2_SO_4_-PVA polymer gel as the electrolyte and separator. (**b**) CV curves of the as-fabricated solid-state device at different scan rates. (**c**) Variation of the specific capacitance of the hybrid hydrogel film electrodes of solid-state device. The inset shows the galvanostatic charge/discharge curves of the device at different current densities. (**d**) CV curves of the rGO-PANI (50%) hybrid-based flexible solid-state supercapacitor at different bending angles with a scan rate of 10 mV/s. The insets show a photograph and a schematic diagram of the flexible solid-state device under the bending state. (**e**) Cycling stability of the solid-state device at a current density of 10 A g^−1^ under bending state. The inset shows the last 10 galvanostatic charge/discharge curves for the device. (**f**) SEM image of the interior microstructure of the dissembled flexible supercapacitor after the cycling process.

To demonstrate the applications of rGO-PANI (50%) hybrid hydrogel film electrodes, flexible solid-state symmetric SCs based on these film electrodes were assembled with H_2_SO_4_-PVA gel electrolyte as separators (Fig. 4a). It’s expected that the continuous porous structures (Fig. 2h,d) of the hydrogel film were able to facilitate efficient ion diffusion between the gel and electrodes. Moreover, the interconnected structure and intimate embedment of PANI NFs in rGO sheets networks can enhance the mechanical properties of SCs.

Figure 4b shows the CV curves of the hybrid hydrogel film based flexible solid-state SCs at different scan rates ranging from 5 to 200 mV s^−1^. The CV curves of SCs display a box-like shape, with pseudocapacitive characteristic peaks attributed by PANI NFs. The current response demonstrated a corresponding increase with the scan rate increase. The peak shifts can be also observed for SCs with the increase of scan rates. It’s important to note that the curve shape of the SCs barely changes with the increase of scan rates and the device maintained the CV shape even at a high scan rate up to 200 mV s^−1^. These characteristics suggest a good capacitive behavior as well as excellent rate capability of SCs.

The GCD curves of flexible solid-state SCs at different current densities ranging from 1 to 20 A g^−1^ maintained an almost triangular shape, as shown in the inset of Fig. 4c, demonstrating their sustainable behaviors even at a high current density. The variations of specific capacitances of the hybrid hydrogel film electrodes of SCs were calculated according to GCD curves of SCs (Supplementary S1.4.3) and shown in Fig. 4c. It’s clear that the specific capacitance decreased with the current density increase. The hydrogel film electrodes of the flexible SCs showed a specific capacitance as high as 844 F g^−1^ at 1 A g^−1^ (211 F g^−1^ for the flexible device), suggesting the efficient infiltration of gel electrolyte into the porous network of hybrid hydrogel film electrodes. Remarkably, the specific capacitance of the hybrid hydrogel film electrodes of SCs is significantly higher than previously reported hybrid film electrodes (Supplementary Table S1). Upon increasing the current density up to 2 and 5 A g^−1^, the specific capacitance remained at 789 and 740 F g^−1^, with the capacity retention of 93.5% and 87.7%, respectively. Even at the very high current density of 20 A g^−1^, the solid-state SCs still exhibited a high specific capacitance of 632 F g^−1^, ~75% of that at 1 A g^−1^.

The SCs exhibited excellent mechanical flexibility in the bending test (the inset of Fig. 4d). The robust hybrid hydrogel films can help the flexible SCs withstand bending at various angles without structural failure and loss of performance. As shown in Fig. 4d, nearly no capacitive changes of CV curves were observed for the devices at various bending angles. Furthermore, the device showed excellent durability performance. The device was characterized by GCD up to 2000 cycles at a high current density of 10 A g^−1^ under 120^o^ bending angle (Fig. 4e) and no loss of specific capacitance was observed. The specific capacitance kept stable at first 500 cycles and increased with the cycle number, finally reached up to 130% of the original specific capacitance. This could be due to the activation process of pseudocapacitive PANI NFs incorporated inside rGO sheet networks. Furthermore, the calculated Coulombic efficiency increased from 94% to nearly 100% after cycling. Such excellent flexibility and capacitive performance of the fabricated device can be attributed to the unique 3D structure of hybrid hydrogel film. In order to figure out the structure stability of electrode materials, the microstructures of the device dissembled after cycling were observed (Fig. 4f). It’s obvious that polymer gel electrolytes had penetrated into the pores of 3D skeletons and ideally protected the whole conductive networks. Hence the favorable interfacial compatibility of electrode with polymer gel electrolyte was achieved, which might also contribute to the outstanding performance^38^.

**Figure 5:**
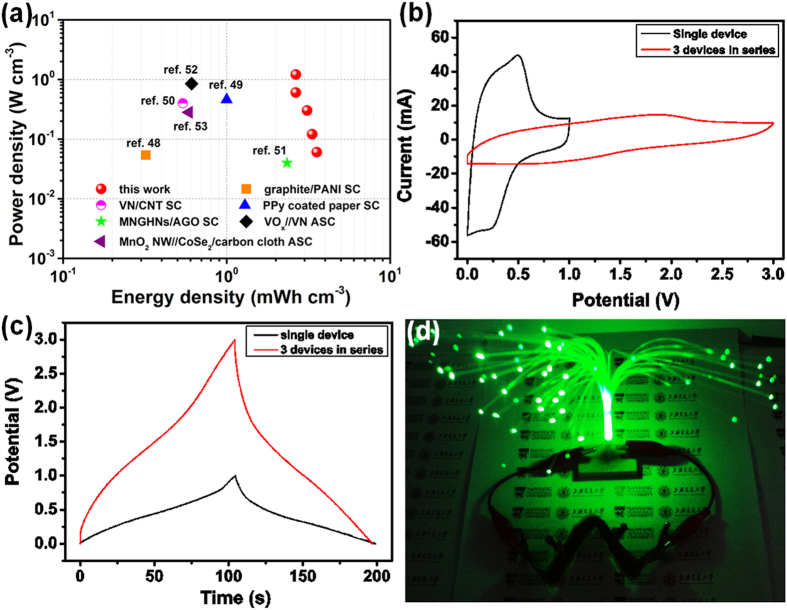
(**a**) The energy and power densities of our flexible SC in comparison with landmark all-solid-state SCs. (**b**) CV curves of a single solid-state supercapacitor and three supercapacitors in series at 100 mVs^−1^. (**c**) Galvanostatic charge/discharge curves of a single solid-state supercapacitor and three supercapacitors in series at 4 mA. (**d**) Photograph of an electronic LED tree (2.5 V) powered by three supercapacitors in series bended with a ‘W’ shape.

Further evaluation of the performance of the solid-state devices was carried out by comparing our SCs with other flexible devices reported to date in Ragone plots (Fig. S11 and Fig. 5a). Figure S11a shows that our SCs exhibited significantly higher gravimetric energy and power densities compared to most of the previously reported flexible SCs. The maximum energy density of 29.3 Wh kg^−1^ (with a power density of 0.5 kW kg^−1^) and power density of 10 kW kg^−1^ (with energy density of 22 Wh kg^−1^) have been achieved by our symmetric SCs based on the total mass of active materials. These values are significantly higher than those of symmetric rGO hydrogel film based SCs (~6.7 Wh kg^−1^ and ~5 kW kg^−1^)^38^, poly(ionic liquid) modified graphene based SCs (6.5 Wh kg^−1^ and 2.5 kW kg^−1^)^39^, MnO_2_/CNT layer based SCs (11.2 Wh kg^−1^ and 5.5 kW kg^−1^)^40^, PANI fiber/rGO based SCs (16.7 Wh kg^−1^ and 986 W kg^−1^)^33^, polypyrrole nanofiber/rGO based SCs (20.6 Wh kg^−1^ and 1280 W kg^−1^)^41^, and even comparable to asymmetric graphene-based devices (1.8 V, 19.7 Wh kg^−1^ and 6.8 kW kg^−1^)^42^ and GF/CNT/MnO2//GF/CNT/PPy based SCs (1.6 V, 22.8 Wh kg^−1^ and 2.7 kW kg^−1^)^43^. Due to the low working voltage of the symmetric device (1 V), the performance of our device is marginally lower than reported asymmetric e-CMG//MnO_2_/e-CMG SCs^44^. We believe the performance will be greatly enhanced through assembling asymmetric devices by applying rGO-PANI hybrid hydrogel film as positive electrodes.

Areal and volumetric energy densities are two most important performance metrics for flexible all-solid-state SCs^34^,^35^. They are calculated and normalized by the whole device including both electrodes, current collectors, gel electrolyte and packaging tape (Supplementary Fig. S11b and Fig. 5a). Our flexible all-solid-state SC, with a high areal capacitance of 0.9 F cm^−2^ (1 A/g or 4.2 mA cm^−2^) and a highly competitive volumetric capacitance of 25.6 F cm^−3^ (1 A g^−1^ or 0.12 A cm^−3^). It can deliver an areal energy density of 0.13 mWh cm^−2^ (2.1 mW cm^−2^ at 4.2 mA cm^−2^), which are substantially higher than those of the reported symmetric cellulose nanofibril/rGO/CNT hybrid aerogels based SC (28.4 μWh cm^−2^ and 9.5 mW cm^−2^)^45^, Manganese (II) phosphate nanosheets-graphene based SC (0.17 μWh cm^−2^ and 46 μW cm^−2^)^46^, and asymmetric rGO/MnO_2_//rGO SCs (35.1 μWh cm^−2^ and 3.8 mW cm^−2^)^47^. Also, the device can deliver a high volumetric energy density of 3.6 mWh cm^−3^ (0.06 W cm^−3^ at 0.12 A cm^−3^), the best values achieved in graphene film-based solid-state symmetric SCs with aqueous polymer gel as electrolyte to date, and much higher than many of recently reported all-solid-state SCs, such as graphite/PANI SC (0.32 mWh cm^−3^ at 0.054 W cm^−3^)^48^, polypyrrole coated paper SC (1 mWh cm^−3^ at 0.27 W cm^−3^)^49^, VN/CNT SC (0.54 mWh cm^−3^ at 0.4 W cm^−3^)^50^, MnOOH/nitrogen-doped graphene SC (2.34 mWh cm^−3^ at 0.04 W cm^−3^)^51^, VO_x_//VN asymmetric SC (0.61 mWh cm^−3^ and 0.85 W cm^−3^)^52^, and MnO_2_ nanowire//CoSe_2_/carbon cloth asymmetric SC (0.588 mWh cm^−3^ at 0.282 W cm^−3^)^53^. It is even comparable to high-energy lithium thin film batteries (1–10 mWh cm^−3^)^54^.

In order to demonstrate the potentials of the flexible SCs, three devices connected in series, as a tandem device, were studied by CV and GCD (Fig. 5b,c). As shown in Fig. 5b,c, the potential window is extended from 1 V for a single device to 3 V for a tandem device. Moreover, the tandem device shows almost unchanged charge/discharge time compared with a single device at the same current density (Fig. 5c). After charged at 3 V, the tandem device bended with a ‘W’ shape can light up an electronic LED tree (2.5 V, Fig. 5d), highlighting the practical potentials of our flexible SCs with the unique 3D structures.

In summary, we demonstrated here a design of high-performance flexible electrodes based on a hierarchical three-dimensional structure. The porous hydrogel films with this unique structure were facilely fabricated through embedment of PANI NFs in continuously interconnected rGO nanosheet network skeletons. With PANI NFs being tightly wrapped inside rGO sheets and subsequently embedded inside of 3D rGO nanosheet frameworks, the resultant films are robust as flexible electrodes. The intimate incorporation of PANI NFs in rGO sheet skeletons and the direct interconnection of all rGO nanosheets in the hybrid electrodes enable rapid electron and ion transport as well as excellent electrochemical behavior. Superior areal and volumetric energy storage capability, high-rate capability as well as excellent cycling stability under bending state without structural failure and loss of performance have been achieved. The integration of PANI NFs in rGO networks meets the requirement of high performance flexible film electrodes and paves an avenue for exploring new structures of hybrids for high-performance flexible energy storage devices.

## Methods

### Preparation of rGO-PANI NF hybrid hydrogel films

Graphene oxide (GO) used here was prepared by the modified Hummers method^28^. PANI NFs were synthesized according to the method reported by Kaner *et al*^55^. In order to disperse PANI NFs in aqueous solution, the synthesized NF acid suspensions were centrifuged at 8000 rpm with deionized water, until the pH of the solution reached ~4. The typical preparation of rGO-PANI NFs hydrogels were illustrated in detail in S1.1. The rGO hydrogel and rGO-PANI NF hybrid hydrogels with different weight ratios of PANI (5%, 10%, 50%, and 80% based on the total weight of GO and PANI, denoted as rGO-PANI (X), X stands for the content of PANI) were fabricated. In order to fabricate hydrogel films, the as-prepared hydrogels were cut into rectangular strips with the thickness of ~2 mm and pressed on the carbon paper substrates under a pressure of ~1 MPa to form thin hydrogel films.

### Fabrication of flexible solid-state SCs

The H_2_SO_4_-PVA gel electrolyte was prepared according to the method reported previously^31^. The fabricated hydrogel films were used directly as the electrodes and poured with H_2_SO_4_-PVA aqueous solution. The hydrogel films with electrolyte were air-dried at room temperature for 12 h to evaporate excess water. Then a pressure of ~1 MPa was applied for 15 min to press the two electrodes together, in order to make sure the polymer gel electrolyte on each electrode combine into one thin separating layer and form an integrated device.

### Characterization

The structures of the hydrogel and film electrodes were characterized by field emission scanning electron microscopy (FE-SEM) and transmission electron microscopy (TEM), the specific surface area measurements, UV-Vis spectrometer, Raman spectrometer, X-ray photoelectron spectrometry (XPS), and Fourier transform infrared (FT-IR) spectrometer (see details in Supplementary S1.2). All the electrochemical experiments, including cyclic voltammetry (CV), galvanostatic charge-discharge measurements (GCD), and impedance spectroscopy measurements (EIS), were carried out at room temperature using a CHI760E workstation. EIS tests were performed over a frequency range from 10^5^ to 10^−2^ Hz at amplitude of 10 mV. As for three-electrode tests, hydrogel films on carbon papers were used directly as the working electrode. Pure PANI NF electrodes were prepared by mixing products with carbon black and polytetrafluoroethylene (mass ratio of 85: 10: 5). The mixture was kneaded and the resultant pastes were pressed onto carbon paper at 10 MPa for 1 min and dried at 80 ^o^C for 12 h for further testing. Platinum foil and saturated calomel electrode (SCE) were used as counter and reference electrode, respectively. 1 M H_2_SO_4_ was used as electrolyte. CV curves and GCD measurements were recorded at the potential range of −0.2 to 0.8 V.

## Additional Information

**How to cite this article**: Hu, N. *et al.* Three-dimensional skeleton networks of graphene wrapped polyaniline nanofibers: an excellent structure for high-performance flexible solid-state supercapacitors. *Sci. Rep.* **6**, 19777; doi: 10.1038/srep19777 (2016).

## Supplementary Material

Supplementary Information
