# Supplementary material for: Three-dimensional skeleton networks of graphene wrapped polyaniline nanofibers: an excellent structure for high-performance flexible solid-state supercapacitors
Source: Sci Rep. 2016 Jan 22;6:19777. doi: 10.1038/srep19777 (PMC4726342; doi:10.1038/srep19777)
Supplement: Supplementary Information [file srep19777-s1.pdf]

Supplementary Information

Three-dimensional skeleton networks of graphene wrapped polyaniline nanofibers: an excellent structure for high-performance flexible solid-state supercapacitors

Nantao Hu,[1,2] Liling Zhang,[1] Chao Yang,[1] Jian Zhao,[2,3] Zhi Yang,[1]* Hao Wei,[1] Hanbin Liao,[2,3] Zhenxing Feng,[4] Adrian Fisher,[5] Yafei Zhang,[1] Zhichuan J. Xu[2,3]*

[1]Key Laboratory for Thin Film and Microfabrication Technology of the Ministry of Education, School of Electronics, Information and Electrical Engineering, Shanghai Jiao Tong University, Dong Chuan Road No.800, Shanghai, 200240, P. R. China;

[2]School of Materials Science & Engineering, Nanyang Technological University, 50 Nanyang Avenue, 639798, Singapore;

[3]Solar Fuels Lab, Nanyang Technological University, 50 Nanyang Avenue, Singapore 639798, Singapore;

[4]Chemical Science and Engineering Division, Argonne National Laboratory, Argonne, IL, 60439, United States;

[5]Department of Chemical Engineering, Cambridge University, Cambridge CB2 3RA, United Kingdom.

E-mails: xuzc@ntu.edu.sg; zhiyang@sjtu.edu.cn

This supplementary file includes:

Materials and Methods
Results and discussion
Figures. S1 to S10
Table S1
References

Table of Contents

S1. **Materials and methods.**……………………………..………………….……….3

S2. **Results and discussion.**·································..………..........................6

S3.**Figure S1:** TEM images of the hybrids.··································..···········7

S4. **Figure S2:** The N_2 adsorption/desorption isotherms of the samples.·····················8

S5. **Figure S3:** Low- and high-magnification SEM images of interior microstructures of freeze-dried hydrogels.··9

S6. **Figure S4:** Low- and high-magnification SEM images of interior microstructures of freeze-dried hydrogel films. ···10

S7. **Figure S5:** XRD patterns of rGO-PANI hybrid hydrogels with different contents of PANI NFs. ··· 11

S8. **Figure S6:** XPS survey curves and C1s XPS profiles of hydrogels.·····················12

S9. **Figure S7:** FTIR spectra of GO, freeze-dired rGO hydrogel and rGO-PANI (50%) hybrid hydrogel powders. ···13

S10. **Figure S8:** CV curves of diffrent electrodes at diffrent scan rates.·····················14

S11. **Figure S9:** Galvanostatic charge/discharge curves of different electrodes at 1 A/g and at different current densities. ··15

S12. **Figure S10:** Low- and high-magnification SEM images of interior microstructures of (a and b) freeze-dried rGO-PANI (50%) hydrogel film after the cycling process, (c and d) PANI NF electrode before cycling, and (e and f) PANI NF electrode after cycling. ··16

S13. **Figure S11:** Ragone plots of the fabricated all-solid-state SC in comparison with recently reported flexible SCs. ···17

S14. **Table S1:** Comparison of the capacitances based on grpahene-PANI hybrid film electrodes. ···18

S15. **Table S2:** Electrochemical performances of various grpahene-based film electrodes in aqueous electrolyte. ···19

S16. **References**···20

S1. Materials and Methods

S1.1 Preparation of rGO-PANI NF hybrid hydrogel films

In a typical preparation of rGO-PANI NFs hydrogel films, 40 mg of GO in 30 mL deionized water were ultrasonicated for 1 h to give a stable suspension. Subsequently, 10 mL of PANI NFs aqueous suspensions with PANI weight ratio of 50 wt% (based on the total weight of GO and PANI) were dipped into GO colloids. After keeping sonication for 30 min, 40 mg of L-cysteine was added and the mixture was allowed to be further sonicated for another 5 min. The resultant suspension was then sealed in a 50 mL Teflon-lined autoclave and maintained at 90 ^{o}C for 12 h. The autoclave was naturally cooled to room temperature, and the as-prepared hydrogel, denoted as rGO-PANI (50%), was taken out with a tweezer and washed by soaking in deionized water 3 times and kept in 1 M H_2SO_4 aqueous solution. The rGO hydrogel and rGO-PANI NF hybrid hydrogels with other weight ratios of PANI (5%, 10%, and 80% based on the total weight of GO and PANI, denoted as rGO-PANI (X), X stands for the content of PANI) were also fabricated according to the method mentioned above.

S1.2 Characterization methods

The morphologies of samples were characterized by scanning electron microscopy (FE-SEM, Carl Zeiss Ultra 55) and transmission electron microscopy (JEM-2100, Japan). The surface area studies of freeze-dried hydrogels were performed on a Micromeritics Tristar 3020 automated gas adsorption system. UV-Vis absorption spectra were conducted on a Perkin-Elmer Lambda 950 UV-Vis-NIR spectrophotometer. Raman scattering was performed on a Renishaw inVia Reflex Raman spectrometer using a 514-nm laser source. X-ray photoelectron spectrometry (XPS) was carried out on a Kratos Axis Ultra DLD using monochromated Al Kα X-ray beams as the excitation source (1486.6 eV). Fourier transform infrared (FT-IR) spectra were recorded on a Bruker (Germany) VERTEX 70 spectrometer (KBr pellets) over a range from 400 to 4000 cm^{-1} with DTGS or MCT as detector.

S1.3 Details of calculation methods for the weight ratios of rGO sheets and PANI NFs in hybrid hydrogels

According to the reference,[S1] the weight ratios of rGO sheets (c_{rGO}) and PANI NFs (c_{PANI}) in the hybrid hybrogels were calculated according to the following equations:

$$c_{\mathrm{PANI}}=\frac{m_{hybrid}-m_{rGO}}{m_{hybrid}}\times 100\% \qquad (1)$$

$$c_{\mathrm{rGO}}=1-c_{\mathrm{PANI}} \qquad (2)$$

where m_{rGO} and m_{hybrid} are the weight of freeze-dried rGO hydrogel and hybrid hydrogels, respectively.

S1.4 Details of calculation methods for the electrochemical (EC) performances of electrodes and devices

S1.4.1 Calculation methods for the EC data of single electrodes tested by three-electrode method:

The specific capacitances of single electodes were tested by three-electrode systems and calculated from their galvanostatic charge/discharge (GCD) curves according to the following equation:

$$C_s = \frac{I \times \Delta t}{\Delta V \times m} \tag{3}$$

where C_s (F/g) is the gravimetric specific capacitance, I is the constant discharging current, Δt is the discharging time, ΔV (V) is the voltage change during the discharging process (excluding voltage drop at the beginning of the discharge), and m is the mass loading of active electrode materials.

The corresponding volumetric capacitances (C_{vol}) of single electrodes were calculated using the following formula:

$$C_{vol} = C_s \times \rho \tag{4}$$

where ρ is the density of hybrid hydrogel films.

S1.4.2 Calculation methods for the enhanced capacitance of rGO-PANI (50%) hydrogel film contributed by synergistic effects:

Based on the specific capacitances of rGO hydrogel film and PANI NFs, the enhanced capacitance of rGO-PANI (50%) hydrogel film contributed by the synergistic effect is calculated according to the following formula:

$$C_{enhanced} = C_{hybrid} - C_{rGO} \times c_{rGO} - C_{PANI} \times c_{PANI} \tag{5}$$

where $C_{enhanced}$ is the enhanced capacitance of rGO-PANI (50%) hydrogel film contributed by the synergistic effect, C_{hybrid} is the capacitance value of rGO-PANI (50%) hydrogel film tested by three-electrode method (921 F/g at 1 A/g in this work), C_{rGO} and C_{PANI} are the capacitances of rGO hydrogel film (128 F/g at 1 A/g in this work) and PANI electrode materials (575 F/g at 1 A/g in this work) respectively, c_{rGO} and c_{PANI} stand for the weight ratios of rGO sheets and PANI NFs, which are calculated to be 31 wt% and 69 wt% respectively (S1.3).

According to the above equation, the enhanced capacitance can be calculated as follows:

$$C_{enhanced} = 921 - 128 \times 31\% - 575 \times 69\% = 485 \text{ F/g}$$

This value reaches 52.7% (485/921×100%) of that of the hybrid hydrogel film, revealing the key role of synergistic effect in the novel 3D structure on improving the final capacitive behavior.

S1.4.3 Calculation methods for the EC data of assembled symmetric supercapacitors (SCs):

Device capacitance (C_{device}) and specific capacitance of individual rGO-PANI (50%) hydrogel film electrode (C_{sp}) were calculated from the slope of the discharge curves of the devices using the following equations:

$$C_{device}=\frac{I\times\Delta t}{m\Delta V} \quad (6)$$

$$C_{sp}=4C_{device}=\frac{4I\times t}{m\times\Delta V} \quad (7)$$

where I is the applied current, m is the whole mass of hybrid materials on both two electrodes, Δt is the discharging time, ΔV is the voltage change during the discharging process (excluding voltage drop at the beginning of the discharge).
The areal capacitance ($C_{areal\text{-}device}$) and volumetric capacitance ($C_{vol\text{-}device}$) of the whole device (normalized by the whole device including active materials, gel electrolyte, current collectors, and packaging tape) were calculated according to the following equations:

$$C_{areal\text{-}device}= C_{device}\times\alpha \quad (8)$$

$$C_{vol\text{-}device}= C_{device}\times\frac{a_{active}}{a_{device}} \quad (9)$$

where α is the areal mass loading of the active material (mg/cm^2) in the device, a_{active} is the thickness of the active materials in the device, a_{device} is the thickness of the whole device.

Gravimetric energy density (E_{wt}, Wh/kg), volumetric energy density (E_{vol}, Wh/L), gravimetric power density (P_{wt}, kW/kg), and volumetric power density (P_{vol}, kW/L) against two electrodes in SCs were calculated based on the following equations:

$$E_{wt}=\frac{1000}{2\times3600}C_{device}\Delta V^2 \quad (10)$$

$$P_{wt}=\frac{E_{wt}}{\Delta t} \quad (11)$$

$$E_{vol}= E_{wt}\times \rho \quad (12)$$

$$P_{vol}= P_{wt}\times \rho \quad (13)$$

where C_{device} is the capacitance of the devices calculated according to equation 6, Δt is the discharging time, ΔV is the operating voltage (obtained from the discharge curve substracted by the voltage drop at the biginning of the discharge).

The areal energy density ($E_{areal\text{-}device}$), areal power density ($P_{areal\text{-}device}$), volumetric energy density ($E_{vol\text{-}device}$), and volumetric power density ($P_{vol\text{-}device}$) of the whole device (normalized by the whole device including active materials, gel electrolyte, current collectors, and packaging tape) were calculated based on following formula:

$$E_{areal\text{-}device}=\frac{1}{2\times3600}C_{areal\text{-}device}\Delta V^2 \quad (14)$$

$$P_{\text{areal-device}} = \frac{E_{\text{areal-device}}}{\Delta t} \quad (15)$$

$$E_{\text{vol-device}} = E_{\text{vol}} \times \frac{a_{active}}{a_{device}} \quad (16)$$

$$P_{\text{vol-device}} = P_{vol} \times \frac{a_{active}}{a_{device}} \quad (17)$$

S2. Results and discussion

S2.1 Surface area characterization and discussion

Brunauer-Emmett-Teller (BET) and Barrett-Joyner-Halenda (BJH) analysis revealed that the freeze-dried rGO hydrogels had a specific surface area of 116 m^2g^{-1} and the pore sizes in the range of 2-50 nm (Figure S2). The specific surface areas of rGO-PANI hybrids varied with different contents of PANI NFs, showing the values of 134, 46, 104, and 63 m^2g^{-1}, respectively, for freeze-dried rGO-PANI (5%), rGO-PANI (10%), rGO-PANI (50%), and rGO-PANI (80%) hydrogels. Considering the inevitable underestimation of specific surface areas due to partial re-stacking of some rGO layers and the fusing of mesopores within hydrogels during the freeze-drying process, the intrinsic surface areas of the wet hydrogels were also determined by employing the methylene blue dye adsorption method.[S1] High specific surface areas of 585, 479, 601, 612, and 375 m^2g^{-1}, for rGO, rGO-PANI (5%), rGO-PANI (10%), rGO-PANI (50%), and rGO-PANI (80%) hydrogels respectively, were achieved based on this approach.

S2.2 The confirmation of the reduction of GO and existence of PANI

The reduction of GO and existence of PANI in the hybrid hydrogels were also confirmed by XPS (Figure S6). Compared with GO, rGO hydrogels exhibited much lower intensity of oxygen functional groups, suggesting the formation of rGO after reduction process (Figure S6a-d).[S1] The appearance of N and C-N characteristic peaks in survey and deconvolution curves revealed the existence of PANI in the hybrid hydrogels (Figure S6e and f). Further confirmation was carried out by FTIR spectra as shown in Fig. S7. The C=O vibration band located at 1732 cm^{-1} was disappeared after hydrothermal reduction of GO.[S2] And the freeze-dried hybrid hydrogel powder showed a series of characteristic peaks of PANI, locating at 1585 and 1504 cm^{-1} (C=C stretching of the quinonoid ring and benzenoid ring respectively), 1308 cm^{-1} (C-N stretching of secondary aromatic amines), and 1232 and 1154 cm^{-1} (C-H bendings of the benzenoid ring and the quinonoid ring respectively).[S3]

S3. Figure S1

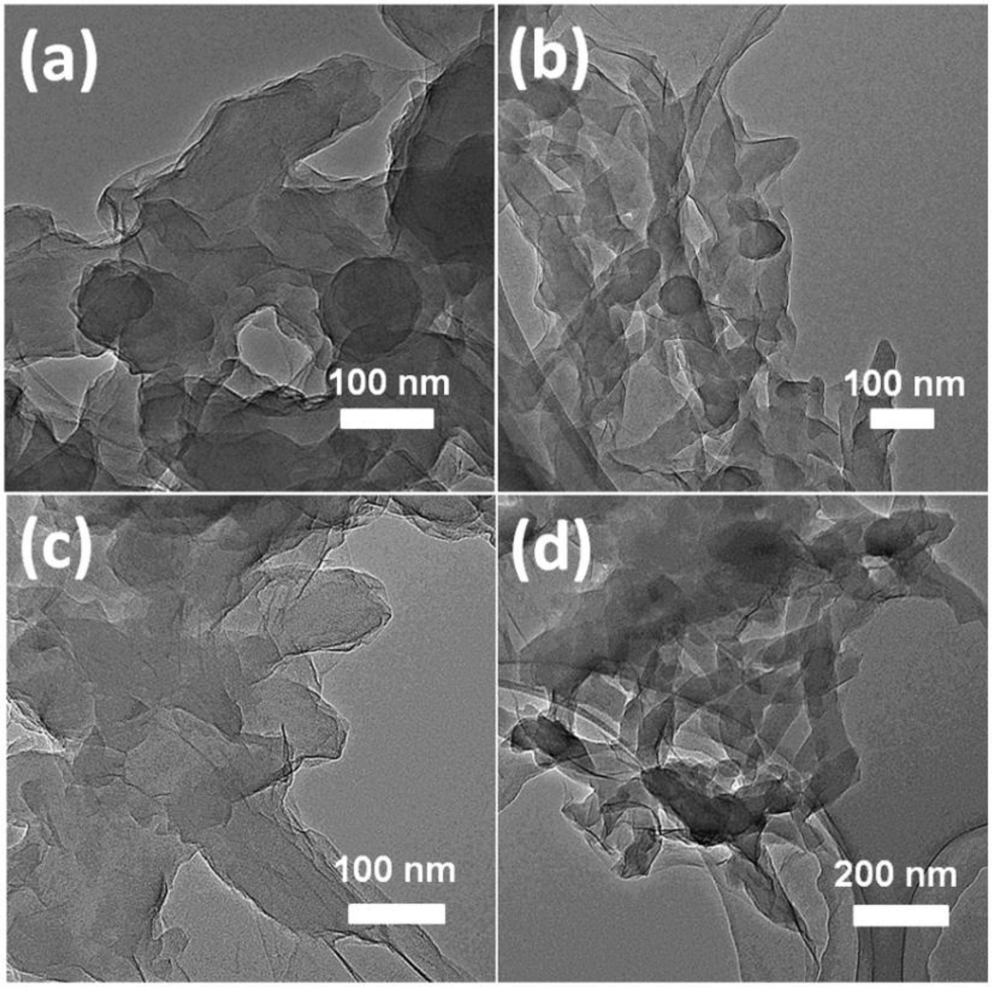

Figure S1. TEM images of the hybrids with different contents of PANI. (a) GO-PANI (5%), (b) GO-PANI (10%), (c) GO-PANI (50%), and (d) GO-PANI (80%) hybrids.

S4. Figure S2

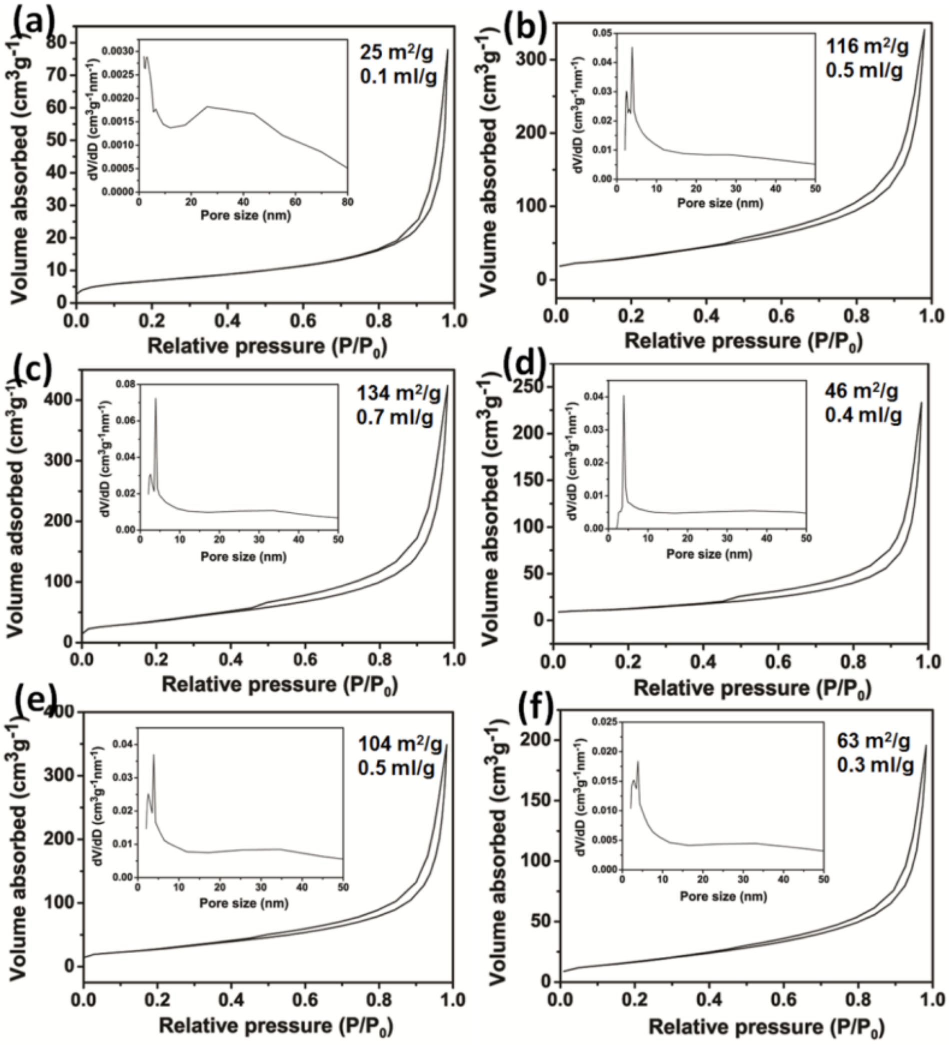

Figure S2. The N_2 adsorption/desorption isotherms of samples. (a) PANI NFs and freeze-dried (b) rGO hydrogel, (c) rGO-PANI (5%) hybrid hydrogel, (d) rGO-PANI (10%) hybrid hydrogel, (e) rGO-PANI (50%) hybrid hydrogel, and (f) rGO-PANI (80%) hybrid hydrogel. The insets show their corresponding pore size distributions.

S5. Figure S3

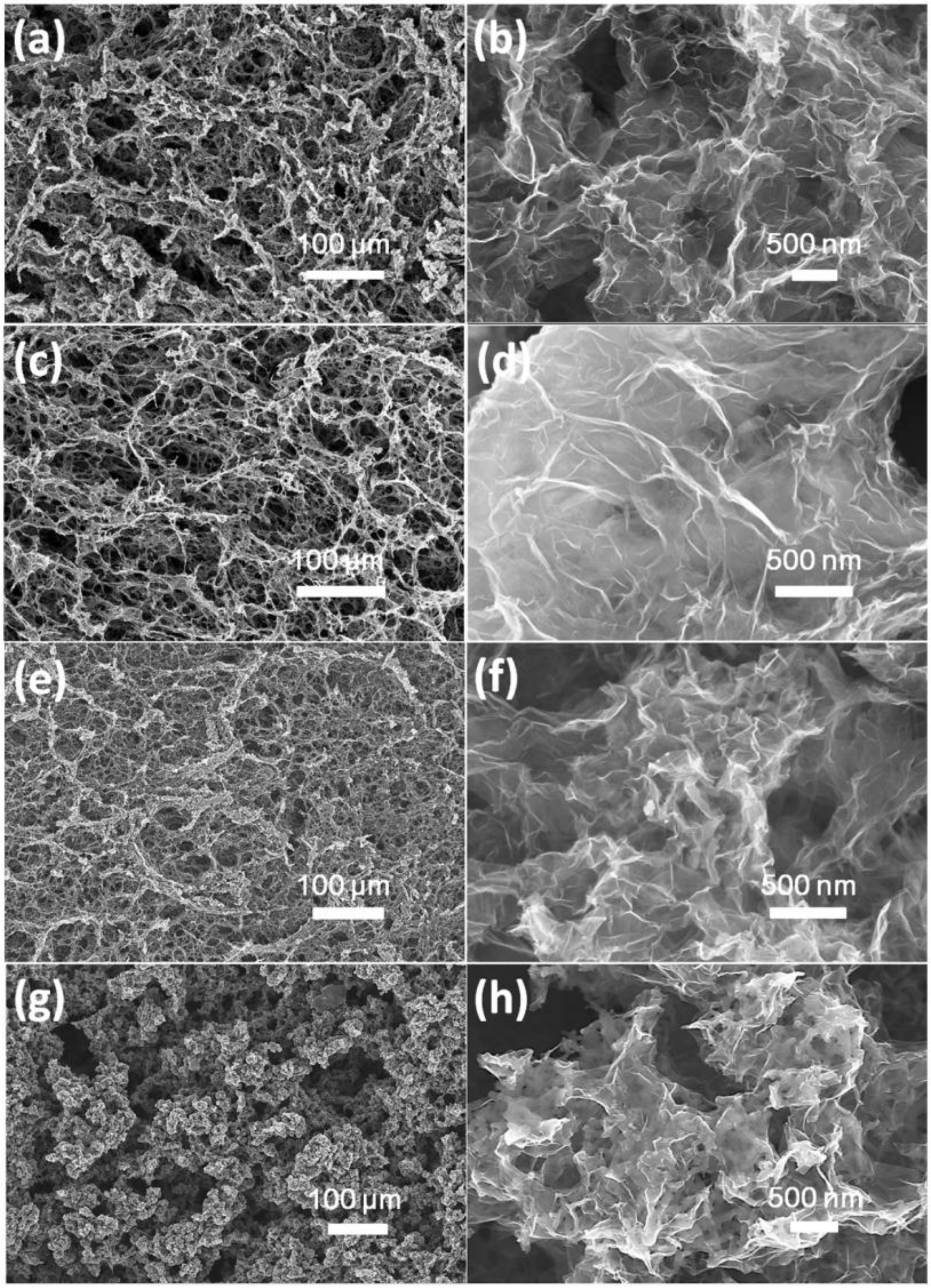

Figure S3. Low- and high-magnification SEM images of interior microstructures of freeze-dried hydrogels: (a and b) rGO hydrogel, (c and d) rGO-PANI (5%) hydrogel, (e and f) rGO-PANI (10%) hydrogel, and (g and h) rGO-PANI (80%) hydrogel.

S6. Figure S4

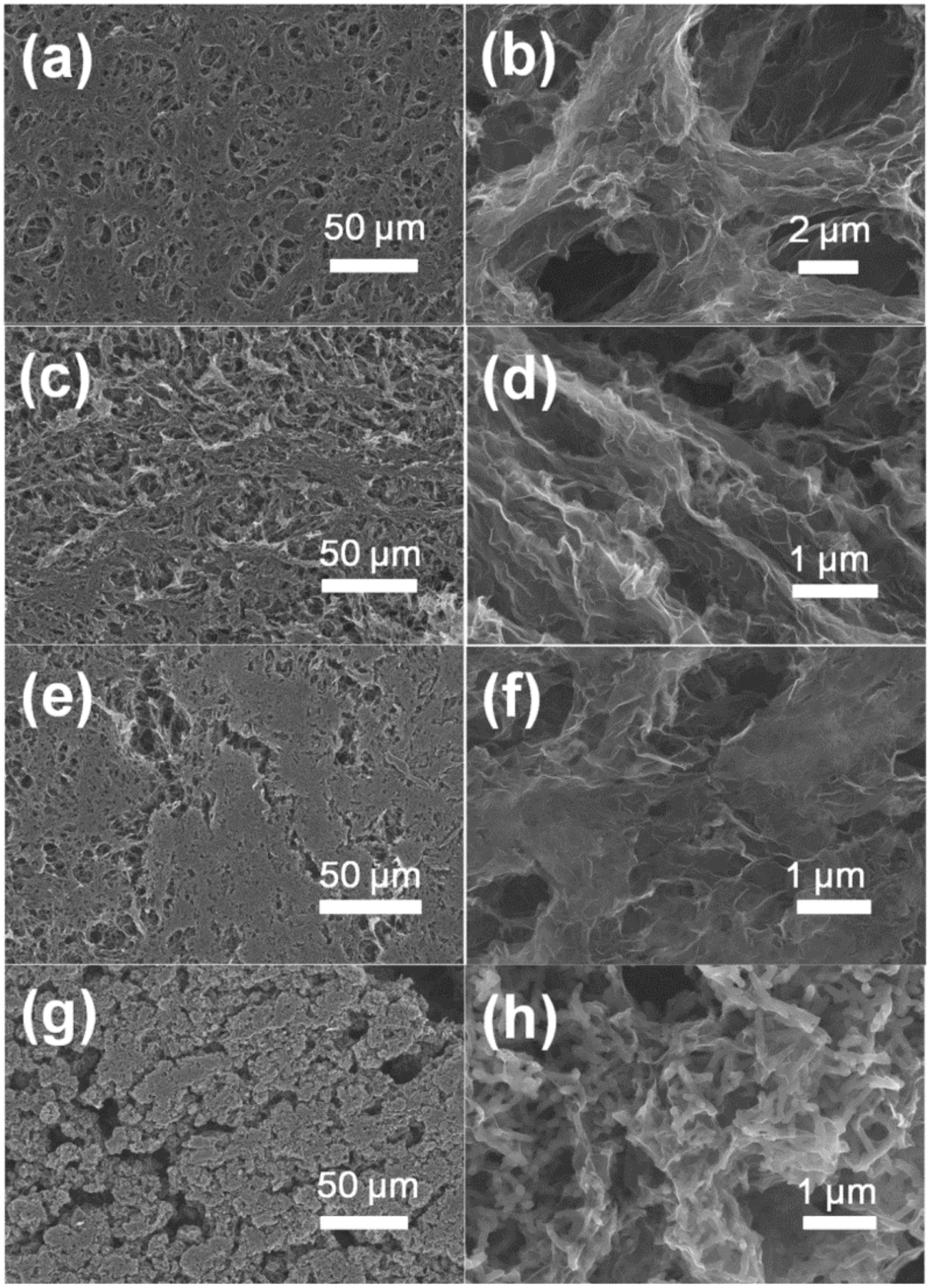

Figure S4. Low- and high-magnification SEM images of interior microstructures of freeze-dried hydrogel films: (a and b) rGO hydrogel film, (c and d) rGO-PANI (5%) hydrogel film, (e and f) rGO-PANI (10%) hydrogel film, and (g and h) rGO-PANI (80%) hydrogel film.

S7. Figure S5

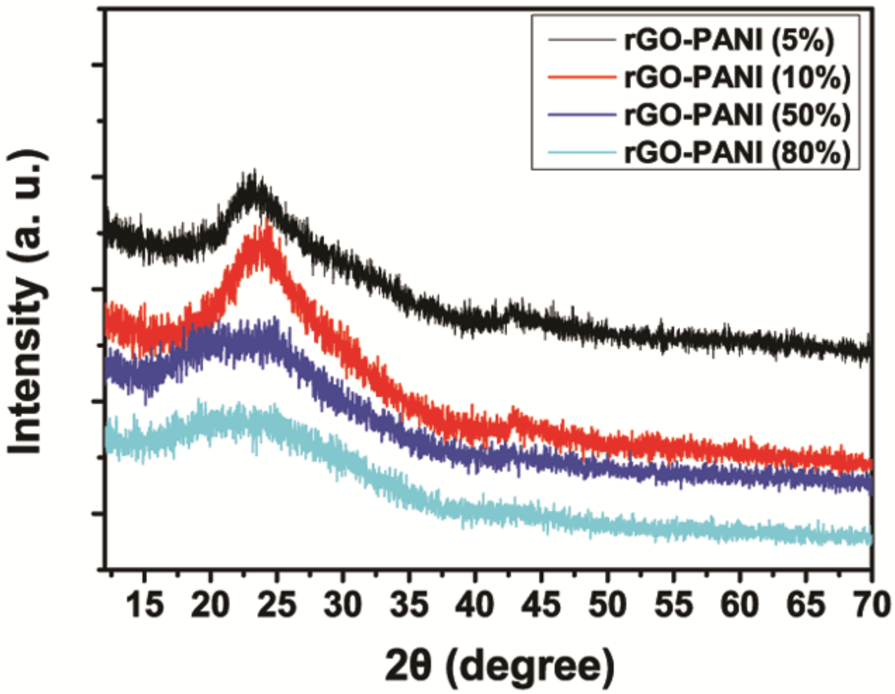

Figure S5. XRD patterns of rGO-PANI hybrid hydrogels with different contents of PANI NFs.

S8. Figure S6

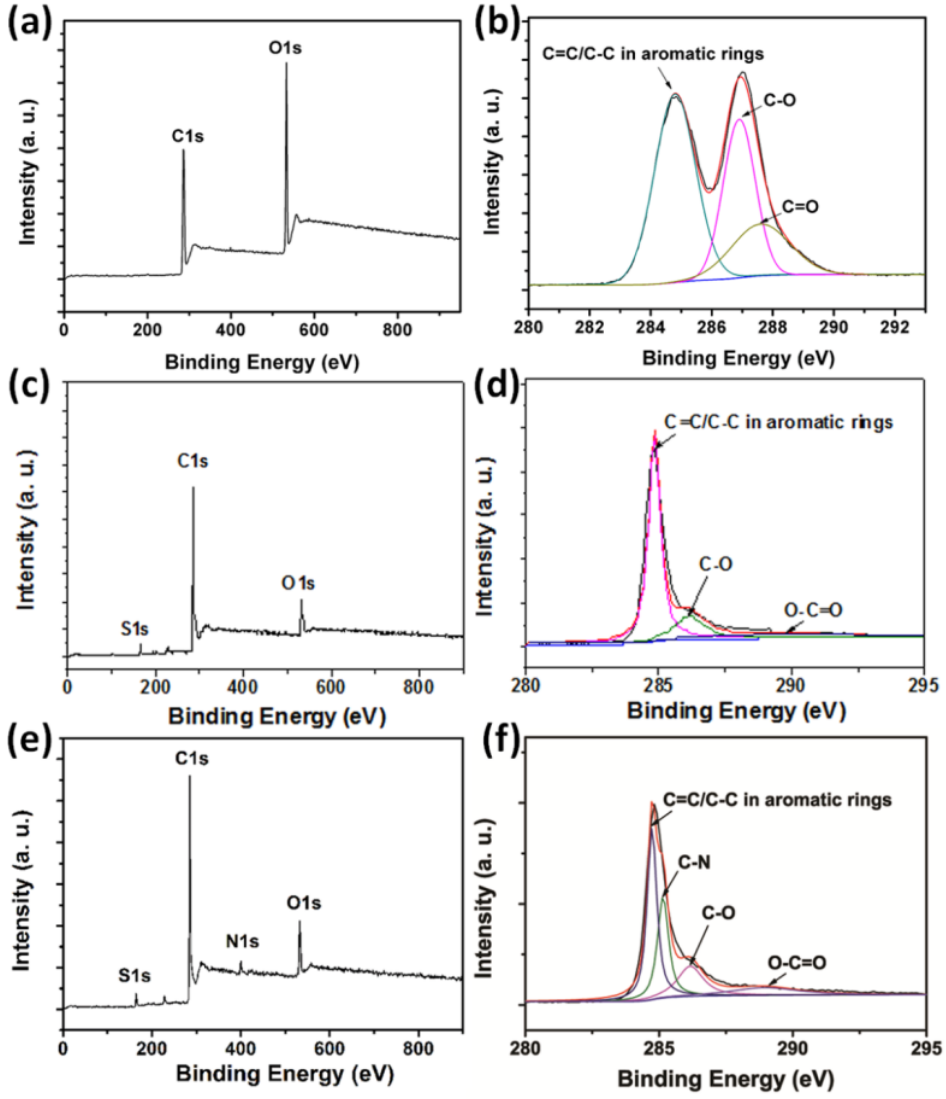

Figure S6. XPS survey curves and C1s XPS profiles of hydrogels: (a and b) GO, (c and d) freeze-dried rGO hydrogel, and (e and f) freeze-dried rGO-PANI (50%) hybrid hydrogel.

S9. Figure S7

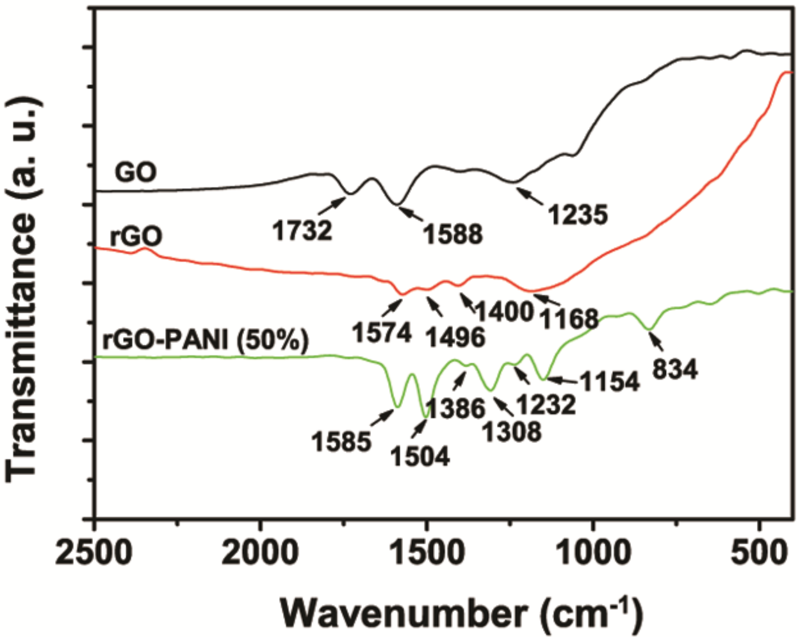

Figure S7. FTIR spectra of GO, freeze-dired rGO hydrogel and rGO-PANI (50%) hybrid hydrogel powders.

S10. Figure S8

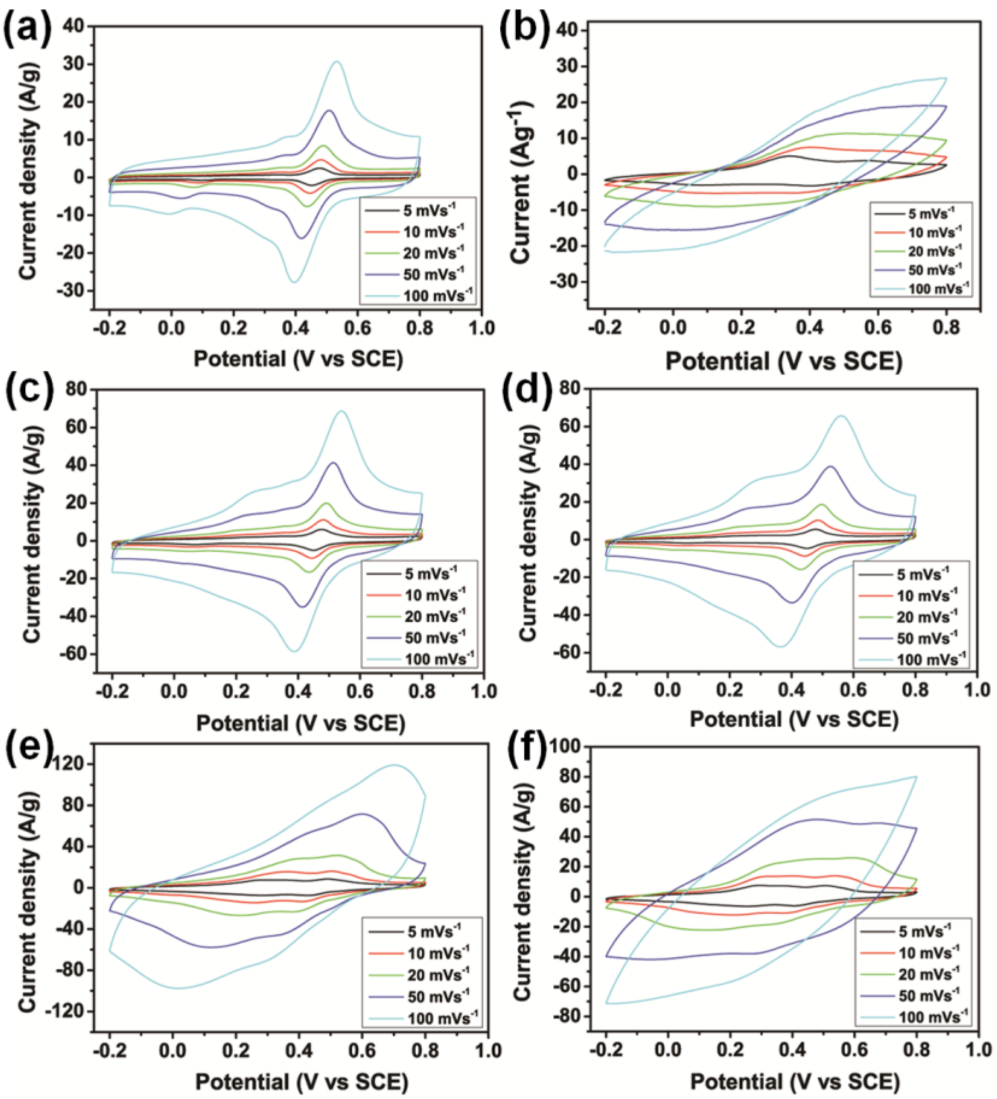

Figure S8. CV curves of diffrent electrodes at diffrent scan rates: (a) rGO hydrogel film, (b) PANI NF, (c) rGO-PANI (5%) hydrogel film, (d) rGO-PANI (10%) hydrogel film, (e) rGO-PANI (50%) hydrogel film, and (f) rGO-PANI (80%) hydrogel film.

S11. Figure S9

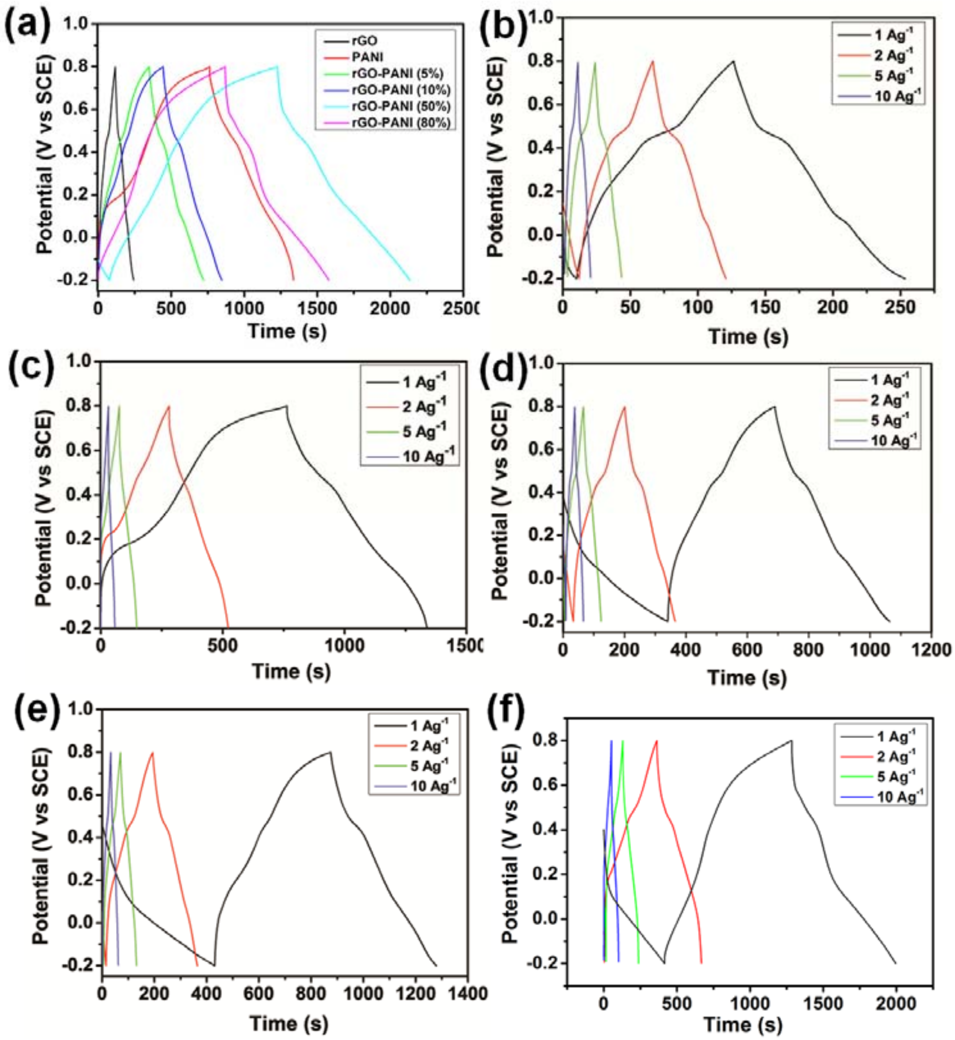

Figure S9. Galvanostatic charge/discharge curves of different electrodes at 1 A/g and at different current densities: (a) all electrodes charged/discharged at 1 A/g, and (b) rGO hydrogel film, (c) PANI NF, (d) rGO-PANI (5%) hybrid hydrogel film, (e) rGO-PANI (10%) hybrid hydrogel film, and (f) rGO-PANI (80%) hybrid hydrogel film electrodes charged/discharged at different current densities.

S12. Figure S10

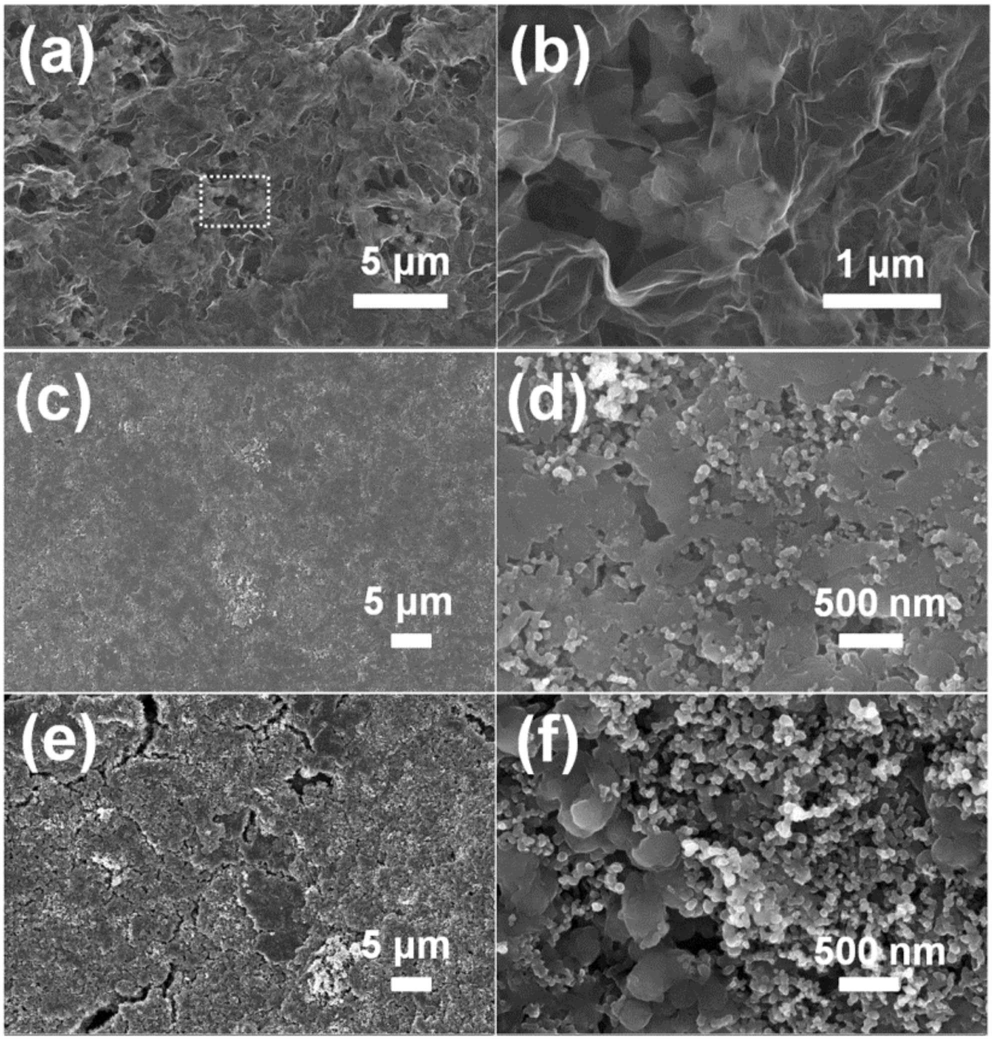

Figure S10. Low- and high-magnification SEM images of interior microstructures of (a and b) freeze-dried rGO-PANI (50%) hydrogel film after the cycling process, (c and d) PANI NF electrode before cycling, and (e and f) PANI NF electrode after cycling.

S13. Figure S11

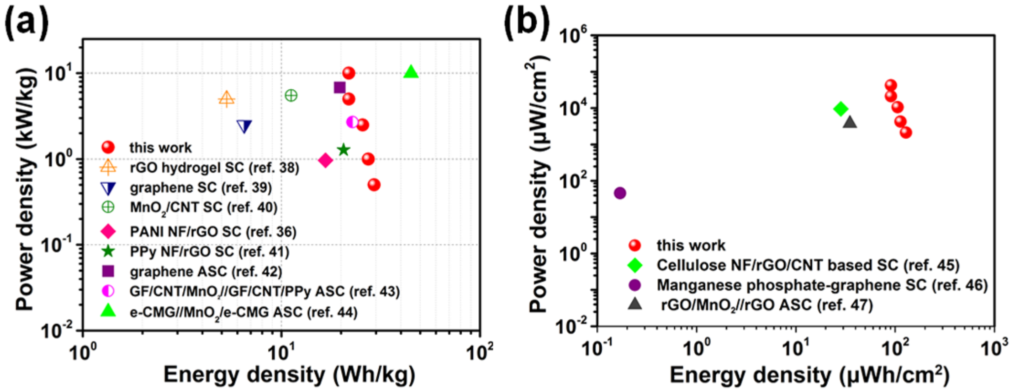

Figure S11. Ragone plots of the fabricated all-solid-state SC in comparison with recently reported flexible SCs. (a) Gravimetric power density vs gravimetric energy density based on the weight of active materials in both electrodes. (b) Areal power density vs areal energy density based on the active area of the device.

S14. Table S1

Table S1 Comparison of the elelctrochemical performance for graphene (GR)-polyaniline (PANI) hybrid film electrodes and SCs

Materials	Mass loading (mg/cm^2)	Testing method	Capacitance		Energy density		Cycle life	Ref
			Gravimetric (F/g)	Volumetric (F/cm^3)	Gravimetric (Wh/kg)	Volumetric (Wh/L)		
GR-PANI composite paper	NA (density of GR paper is 0.2 g/cm^3)	3-electrode GCD	763 (1 A/g)	NA	NA	NA	82% (1000 cycles at 5 A/g)	[S4]
GR/PANI composite film	With the thickness of 20-30 nm	3-electrode GCD	640 (0.1 A/g)	NA	NA	NA	90% (1000 cycles at 0.1 A/g	[S5]
GR-PANI hybrid film	27.7 $\mu g/cm^2$	3-electrode GCD	375.2 (0.5 A/g)	NA	30.34	NA	90.7% (500 cylcles at 3 A/g)	[S6]
GR/PANI composite paper	4.59-6[a]	3-electrode CV	233 (2 mV/s)	135 (2 mV/s)	NA	NA	No loss (1500 cycles at 50 mV/s)	[S7]
GR-PANI hybrid paper	0.786	3-electrode GCD	489 (0.4 A/g)	NA	NA	NA	96% (500 cycles at 0.4 A/g)	[S8]
GR/PANI NF films	NA	3-electrode GCD	210 (0.3 A/g)	160 (0.3 A/g)	19	NA	94% (800 cycles at 3 A/g)	[S9]
Layered PANI/GR film	1.42	3-electrode GCD	384 (0.5 A/g)	NA	NA	NA	84% (1000 cycles at 2 A/g)	[S10]
PANI/reduced GR oxide film	~2	3-electrode GCD	431 (0.45 A/g)	NA	NA	NA	74% (500 cycles at 0.45 A/g)	[S11]
3D porous GH-PANI nanocomposite/GR paper	NA	3-electrode GCD	864 (1 A/g)	NA	NA	NA	96% (1000 cycles at 8 A/g)	[S12]
LBL GR/PANI bilayers	15 bilayers (nm scale)	3-electrode GCD	NA	584 (3 A/cm^3)	NA	NA	56.5% (1000 cycles at 3 A/cm^3)	[S13]
GR/PANI/CNT ternary composite film	5	3-electrode GCD	569 (0.1 A/g)	188 (0.1 A/g)	79	26.1	96% (5000 cycles at 3 A/g)	[S14]
GR-PANI-PA	3	2-electrode GCD	547	122	NA	NA	90% (4000 cycles at 5 A/g)	[S15]
SC based on 3D GR-PANI NW array	0.65×2	2-electrode GCD	790 (1 A/g)	205.4	17.6 (two electrodes)	NA	80% (5000 clycles at 1 A/g)	[S16]
Sandwich-type GR/PANI/GR paper	NA	3-electrode GCD	581 (1 A/g)	NA	NA	NA	85% (10000 cycles at 10 A/g)	[S17]
**Flexible 3D hydrogel films based on rGO wrapped PANI NFs**	**2.1**	**3-electrode GCD**	**921 (1 A/g)**	**391 (1 A/g)**	**128**	**54.3**	**85% (1000 cycles at 10 A/g)**	**This work**
**Flexible solid-state SCs based on 3D hybrid hydrogel film electrodes**	**2.1×2**	**2-electrode GCD**	**844 (1 A/g)**	**358 (1 A/g)**	**117.2 (one electrode) 29.3 (two electrodes)**	**49.7 (one electrode) 12.4 (two electrodes)**	**No loss (2000 cycles at 10 A/g)**	**This work**

[a] The mass loading is calculated by the density of hybrid papers (0.51-0.58 g/cm^3) multiply by the thickness of hybrid papers (90-104 μm) reported in reference S7.

S15. Table S2

Table S2 Electrochemical performances of various graphene-based film electrodes in aqueous electrolyte.

Materials	Mass loading (mg/cm^2)	Testing method	Capactitance		Energy density		Cycle life	Ref
			Gravimetric(F/g)	Volumetric (F/cm^3)	Gravimetric (Wh/kg)	Volumetric (Wh/L)		
Urea reduced GO	4.5	3-electrode GCD	255 (1 A/g)	196 (0.5 A/g)	NA	NA	93% (1200 cycles at 3 A/g)	[S18]
Holey GR	1	2-electrode GCD	310 (1 A/g)	NA	NA	NA	95% (20,000 cycles at 25 A/g	[S19]
LBL heteroatom-doped GR films	Thickness at nm scale	2-electrode CV	NA	488 (10 mV/s)	NA	16.9	95.2% (100,000 cylcles at 500 V/s)	[S20]
GR/PVP composite film	NA	2-electrode GCD	179.4 (1 A/g)	71.8 (1 A/g)	NA	NA	80% (10,000 cycles at 100 A/g)	[S21]
GR-$Ni(OH)_2$ hybrid film	3	3-electrode GCD	573 (0.2 A/g)	655 (0.2 A/g)	NA	NA	No loss (20,000 cycles at 10 mV/s)	[S22]
GR nanomesh-CNT hybrid film	0.5	3-electrode CV	294 (5 mV/s)	331 (5 mV/s)	NA	26	NA	[S23]
LBL GR- Fe_3O_4 layers	Multilayer with thickness at nm scale	3-electrode CV	NA	280 (10 mV/s)	NA	NA	93% (1000 cycles at 100 mV/s)	[S24]
GR-Polypyrrole films	0.38-2.34	3-electrode CV	285 (0.5 A/g)	NA	NA	NA	74% (800 cycles at 2 A/g)	[S25]
RuO_2/PEDOT:PSS/GR electrode	3	3-electrode GCD	820 (0.5 A/g)	NA	73	NA	81.5% (1000 cycles at 0.5 A/g)	[S26]
GR-Polypyrrole composite	Submicrometer thick	3-electrode GCD	424 (1 A/g)	NA	NA	NA	NA	[S27]
GR/PANI/CNT composite film	1.8	3-electrode GCD	569 (0.1 A/g)	188	NA	NA	96% (5000 cycles at 3 A/g)	[S28]

S16. References

References

[S1] Xu, Y., Lin, Z., Huang, X., Wang, Y., Huang, Y. and Duan, X., Functionalized graphene hydrogel-based high-performance supercapacitors. *Adv. Mater.* **25**, 5779-5784 (2013).

[S2] Hu, N., Wang, Y., Chai, J., Gao, R., Yang, Z., Kong, E. S. W. and Zhang, Y., Gas sensor based on p-phenylenediamine reduced graphene oxide. *Sensor. Actuat. B-Chem.* **163**, 107-114 (2012).

[S3] Mishra, A. K. and Ramaprabhu, S., Functionalized graphene-based nanocomposites for supercapacitor application. *J. Phys. Chem. C* **115**, 14006-14033 (2011).

[S4] Cong, H. P., Ren, X. C., Wang, P. and Yu, S. H., Flexible graphene-polyaniline composite paper for high-performance supercapacitor. *Energy Environ. Sci.* **6**, 1185-1191 (2013).

[S5] Feng, X. M., Li, R. M., Ma, Y. W., Chen, R. F., Shi, N. E., Fan, Q. L. and Huang, W., One-step electrochemical synthesis of graphene/polyaniline composite film and its applications. *Adv. Funct. Mater.* **21**, 2989-2996 (2011).

[S6] Lee, T., Yun, T., Park, B., Sharma, B., Song, H. K. and Kim, B. S., Hybrid multilayer thin film supercapacitor of graphene nanosheets with polyaniline: importance of establishing intimate electronic contact through nanoscale blending. *J. Mater. Chem.* **22**, 21092-21099 (2012).

[S7] Wang, D. W., Li, F., Zhao, J., Ren, W., Chen, Z. G., Tan, J., Wu, Z. S., Gentle, I., Lu, G. Q. and Cheng, H. M., Fabrication of graphene/polyaniline composite paper via in situ anodic electropolymerization for high-performance flexible electrode. *ACS Nano* **3**, 1745-1752 (2009).

[S8] Yan, X., Chen, J., Yang, J., Xue, Q. and Miele, P.,Fabrication of free-standing, electrochemically active, and biocompatible graphene oxide-polyaniline and graphene-polyaniline hybrid papers. *ACS Appl. Mater. Interfaces* **2**, 2521-2529 (2010).

[S9] Wu, Q., Xu, Y. X., Yao, Z. Y., Liu, A. R. and Shi, G. Q., Supercapacitors based on flexible graphene nanofiber composite films. *ACS Nano* **4**, 1963-1970 (2010).

[S10] Tong, Z., Yang, Y., Wang, J., Zhao, J., Su, B. L., Li, Y., Layered polyaniline/graphene film from sandwich-structured polyaniline/graphene/polyaniline nanosheets for high-performance pseudosupercapacitors. *J. Mater. Chem. A* **2**, 4642-4651 (2014).

[S11] Kim, M., Lee, C., Jang, J., Fabrication of highly flexible, scalable, and high-performance supercapacitors using polyaniline/reduced graphene oxide film with enhanced electrical conductivity and crystallinity. *Adv. Funct. Mater.* **24**, 2489-2499 (2014).

[S12] Chi, K., Zhang,Z., Xi, J., Huang, Y., Xiao, F., Wang, S., Liu, Y., Freestanding graphene paper supported three-dimensional porous graphene-polyaniline nanocomposite synthesized by inkjet printing and in flexible all-solid-state supercapacitor. *ACS Appl. Mater. Interfaces* **6**, 16312-16319 (2014).

[S13] Sarker, A. K., Hong, J. D., Layer-by-layer self-assembled multilayer films composed of graphene/polyaniline bilayers: high-energy electrode materials for supercapacitors. *Langmuir* **28**, 12637-12646 (2012).

[S14] Lu, X., Dou, H., Yang, S., Hao, L., Zhang, L., Shen, L., Zhang, F., Zhang, X., Fabrication and electrochemical capacitance of hierarchical graphene/polyaniline/carbon nanotube ternary composite film. *Electrochim. Acta* **56**, 9224-9232 (2011).

[S15] Sekar, P., Anothumakkool, B., Kurungot, S., 3D polyaniline porous layer anchored pillared graphene sheets: enhanced interface joined with high conductivity for better charge storage applications. *ACS Appl. Mater. Interfaces* **7**, 7661-7669 (2015).

[S16] Yu, P., Zhao, X., Huang, Z., Li,Y., Zhang, Q., Free-standing three-dimensional graphene and polyaniline nanowire arrays hybrid foams for high-performance flexible and lightweight supercapacitors. *J. Mater. Chem. A* **2**, 14413-14420 (2014).
[S17] Xiao, F., Yang, S., Zhang, Z., Liu, H., Xiao, J., Wan, L., Luo, J., Wang, S., Liu, Y., Scalablesynthesis of freestanding sandwich-structured graphene/polyaniline/graphene nanocomposite paper for flexible all-solid-state supercapacitor. *Sci. Rep.* **5**, 9359 (2015).
[S18] Lei, Z., Lu, L., Zhao, X. S., The electrocapacitive properties of graphene oxide reduced by urea. *Energy Environ. Sci.*, , 5, 6391-6399 (2012).
[S19] Xu,Y., Lin, Z., Zhong, X., Huang, X., Weiss, N. O., Huang, Y., Duan, X., Holey graphene frameworks for highly efficient capacitive energy storage. *Nat. Commun.* **5**, 4554 (2014).
[S20] Wu, Z. S., Parvez, K., Winter, A., Vieker, H., Liu, X., Han, S., Turchanin, A., Feng, X., Müllen, K., Layer-by-layer assembled heteroatom-doped graphene films with ultrahigh volumetric capacitane and rate capability for micro-supercapacitors. *Adv. Mater.* **26**, 4552-4558 (2014).
[S21] Huang, L., Li, C., Shi, G., High-performance and flexible electrochemical capacitors based on graphene/polymer composite films. *J. Mater. Chem. A* **2**, 968-974 (2014).
[S22] Li, M., Tang, Z., Leng, M., Xue, J., Flexible solid-state supercapacitor basedon graphene-based hybrid films. *Adv. Funct. Mater.* **24**, 7495-7502 (2014).
[S23] Jiang, L., Sheng, L., Long, C., Fan, Z., Densely packed graphene nanomesh-carbon nanotube hybrid film for ultra-high volumetric performance supercapacitors. *Nano Energy* **11**, 471-480 (2015).
[S24] Park, M., Kim, Y., Ko, Y., Cheong, S., Ryu, S. W., Cho, J., Amphiphilic layer-by-layer assembly overcoming solvent polarity between aqueous and nonpolar media. *J. Am. Chem. Soc.* **136**, 17213-17223 (2014).
[S25] Liu, A., Li, C., Bai, H., Shi, G., Electrochemical deposition of polypyrrole/sulfonated graphene composite films. *J. Phys. Chem. C* **114**, 22783-22789 (2010).
[S26] Cho, S., Kim, M., Jang, J., Screen-printable and flexible RuO2 nanoparticle-decorated PEDOT:PSS/graphene nanocomposite with enhanced electrical and electrochemical performances for high-capacity supercapacitor. *ACS Appl. Mater. Interfaces* **7**, 10213-10227 (2015).
[S27] Chang, H. H., Chang, C. K., Tsai, Y. C., Liao, C. S., Electrochemically synthesized graphene/polypyrrole composites and their use in supercapacitor. *Carbon* **50**, 2331-2336 (2012).
[S28] Lu, X., Dou, H., Yang, S., Hao, L., Zhang, L., Shen, L., Zhang, F., Zhang, X., Fabrication and electrochemical capacitance of hierarchical graphene/polyaniline/carbon nanotube ternary composite film. *Electrochim. Acta*, 2011, 56, 9224-9232.
